# Delicate Role of PD-L1/PD-1 Axis in Blood Vessel Inflammatory Diseases: Current Insight and Future Significance

**DOI:** 10.3390/ijms21218159

**Published:** 2020-10-31

**Authors:** Priya Veluswamy, Max Wacker, Maximilian Scherner, Jens Wippermann

**Affiliations:** Heart Surgery Research, Department of Cardiothoracic Surgery, Faculty of Medicine, Otto-von Guericke University, D-39120 Magdeburg, Germany; max.wacker@med.ovgu.de (M.W.); maximilian.scherner@med.ovgu.de (M.S.); jens.wippermann@med.ovgu.de (J.W.)

**Keywords:** programmed death-1 (PD-1), PD-L1, PD-L2, coronary artery disease, atherosclerosis and blood vessel inflammatory diseases

## Abstract

Immune checkpoint molecules are the antigen-independent generator of secondary signals that aid in maintaining the homeostasis of the immune system. The programmed death ligand-1 (PD-L1)/PD-1 axis is one among the most extensively studied immune-inhibitory checkpoint molecules, which delivers a negative signal for T cell activation by binding to the PD-1 receptor. The general attributes of PD-L1’s immune-suppressive qualities and novel mechanisms on the barrier functions of vascular endothelium to regulate blood vessel-related inflammatory diseases are concisely reviewed. Though targeting the PD-1/PD-L1 axis has received immense recognition—the Nobel Prize in clinical oncology was awarded in the year 2018 for this discovery—the use of therapeutic modulating strategies for the PD-L1/PD-1 pathway in chronic inflammatory blood vessel diseases is still limited to experimental models. However, studies using clinical specimens that support the role of PD-1 and PD-L1 in patients with underlying atherosclerosis are also detailed. Of note, delicate balances in the expression levels of PD-L1 that are needed to preserve T cell immunity and to curtail acute as well as chronic infections in underlying blood vessel diseases are discussed. A significant link exists between altered lipid and glucose metabolism in different cells and the expression of PD-1/PD-L1 molecules, and its possible implications on vascular inflammation are justified. This review summarizes the most recent insights concerning the role of the PD-L1/PD-1 axis in vascular inflammation and, in addition, provides an overview exploring the novel therapeutic approaches and challenges of manipulating these immune checkpoint proteins, PD-1 and PD-L1, for suppressing blood vessel inflammation.

## 1. Introduction

The term “vasculitides” is a collective designation referring to any vascular-related ailments where inflammation commences, propels and advances at different zones of the blood vessel wall and the degree of inflammation is considered to be the principal risk factor in promoting the disease severity and the outcomes [1,2]. The general etiology behind blood vessel inflammatory diseases is that they could occur either as a primary idiopathic disease with genetic predispositions or as a secondary response to underlying diseases (e.g., chronic infections, autoimmune reactions, diabetes and metabolic syndrome) [3,4]. Primarily, inflammatory vascular diseases are classified based on the size of the vessels that are being afflicted, including: (i) Takayasu’s arteritis and temporal arteritis (also known as giant cell arteritis) affecting the large-sized vessels [5,6,7]; (ii) Bürger’s disease, Kawasaki disease and polyarthritis nodosa affecting the medium-sized vessels [8,9,10]; (iii) Behcet’s syndrome, granulomatosis with polyangiitis (formerly known as Wegener’s granulomatosis), Henoch-Schönlein purpura, Churg-Strauss vasculitis, cryoglobulinemic vasculitis and microscopic polyangiitis, affecting the small-sized vessels [11,12,13]. Basically, an inflammation-driven narrowing of the arterial lumen can lead to a condition called atherosclerosis, where predominant lipid deposits with various immune cells occur to form a fatty white streak called “plaque” [14]. Such an atherosclerotic inflammation is pronounced to the blood vessels at certain specific sites that cause damage to the organs that are located in proximity to the affected arteries and these secondary vascular diseases are commonly grouped under “cardiovascular diseases (CVDs)” [15]. These include (i) coronary artery disease (CAD), where inflammatory plaques occur in the coronary arteries, which lessens the oxygen supply to the heart muscle and leads to myocardial infarction (MI) [16], (ii) peripheral artery occlusion disease (PAOD), where an inflammation occurs at extremities (such as limbs) and therefore prevails in the regions that are distal from the heart [17], and (iii) carotid stenosis (CS), where inflamed lesions occur in the carotid arteries of neck, which hinders the supply of oxygenated blood to the brain, leading to stroke [18]. Thus, the ultimate prevailing spots for the advancement of atherosclerosis are coronary arteries, popliteal (limb) arteries and carotid arteries and these are henceforth considered as significant underlying factors for the development of CAD, PAOD and CS [14,19]. In contrast to the narrowed vessels, excessive inflammatory reactions can also lead to a localized dilation of blood vessels (such as the aorta), causing structural deformity, which is commonly termed as an aneurysm [20]. Besides the primary blood vessel inflammatory diseases, the secondary vascular diseases are the major classified determinants for atherosclerosis in western countries, where the development of CVD is tightly linked to (i) the current industrialized economy that demands sedentary jobs and technology-driven culture demanding for longer work hours resulting in lack of physical and recreational activities, (ii) intake of a high-calorie diet with saturated fats and refined sugars, and (iii) change in the lifestyle, in particular, cigarette smoking habits irrespective of gender [21]. These aforementioned risk factors in the modern world are majorly responsible for atherogenesis and other associated metabolic disturbances such as obesity, hypertension and type 2 diabetes mellitus which prevail in people with CVD [22,23]. Though we summarized the involvement of PD-1 and PD-L1 or PD-L2 molecules in the pathogenesis of the different primary vasculitides (Table 1), the key content of this review is majorly subjected to a deep-seated awareness and a knowledge acquisition on the role of the PD-L1/PD-1 axis in various pathomechanistic and clinical aspects of atherosclerotic blood vessel inflammatory diseases—in particular, CAD, PAOD, CS, MI and stroke.

Costimulatory molecules (nowadays known as immune checkpoint molecules) are the antigen-independent generator of secondary signals that aid in maintaining the homeostasis of the immune system [24,25]. These immune checkpoint molecules are broadly classified into three family members—the Immunoglobulin (Ig) superfamily, the tumor necrosis factor (TNF) and TNF receptor (TNFR) superfamily and the newly included T cell Immunologlobulin Mucin (TIM) superfamily. Based on their functional attributes, the checkpoint molecules can be either stimulatory or inhibitory. The most extensively studied immune checkpoint molecule pairs are CD28/CTLA-4, expressed on T cells, interacting with CD80/CD86, expressed on antigen-presenting cells (APCs), where the interaction between CD28 and CD80/CD86 activates T cell responses, whereas the high-affinity binding between CTLA-4 and CD80/CD86 inhibits the activated T cell responses. Thereafter, several other well-known interacting pairs were thoroughly investigated, including: (i) CD27-CD70; (ii) ICOS-ICOS-L; (iii) CD40-CD40L; (iv) OX-40-OX-40L; (v) GITR-GITRL, as examples of stimulatory checkpoint molecules and (i) PD-1-PD-L1 and PD-L2; (ii) Tim-3-Galectin-9; (iii) LAG-3-FGL1; (iv) BTLA-HVEM; (v) B7-H3-unknown receptor, as examples of inhibitory checkpoint molecules [26,27,28].

## 2. Overview of General Biological Activities of PD-L1 and PD-1 Molecules

Besides the CD28/CTLA-4 immune checkpoint pair, the interactions between PD-1 and PD-L1 and PD-L2 ligands were the most extensively studied molecules which has led to a profound knowledge and understanding of manipulating various disease settings, with cancer being the paramount setting [38]. This led to a ground-breaking interventional approach for cancer therapy, where many cancer patients who were refractory to conventional chemotherapies benefited from PD-1 blockades and thereby the PD-L1/PD-1 axis blockade finally culminated in the Nobel Prize award in physiology and medicine being awarded in the year 2018 for a revolutionized accomplishment in the field of cancer therapeutics [39,40].

The receptor, PD-1, was identified to be induced mainly on activated T cells and was initially reported to be involved in programmed cell death [41]. Despite T cells, the expression of PD-1 was also evident on APCs, monocytes and macrophages [42,43]. PD-L1 was thereafter consecutively reported to be a chief ligand for PD-1 which was shown to deliver a negative signal to the PD-1-expressing cells [44,45]. One year later, PD-L2 was discovered to be the second ligand for PD-1, which inhibited T cell activation and minimized cytokine production during low as well as high antigenic concentrations, respectively [45]. While PD-L1 is broadly expressed on haemopoietic and non-haemopoietic cells, the expression of PD-L2 is limited to professional antigen-presenting cells (APCs) [46]. Several PD-1 knockout experiments on different mouse models have affirmed the role of PD-1 in maintaining peripheral tolerance [47,48]. The cell membrane-bound PD-1 imparts the signal through its cytoplasmic tail which contains two tyrosine-based structural motifs, an immunoreceptor tyrosine-based inhibitory motif (ITIM) (V/L/I/XpYXX/L/V) and an immunoreceptor tyrosine-based switch motif (ITSM) (TXpYXXV/I). Though both the motifs act as docking sites for the recruitment of Src homology region 2 domain-containing phosphatase-1 (SHP-1) and SHP-2, mutational studies have revealed that ITSM phosphotyrosine, which preferentially recruits SHP-2, is crucial for the key inhibitory function of PD-1 resulting in down-modulation of intracellular signaling. However, the exact molecular mechanisms of PD-1-mediated inhibition of T lymphocytes are still being scrutinized and are therefore under investigation [49,50]. The signal becomes functional mainly when the PD-1 molecules colocalize with CD3/CD28 receptors, where simultaneous TCR (T Cell Receptor) signaling is considered pivotal for SHP-2 to dephosphorylate the TCR activation molecules ZAP70 and CD3δ, leading to the repression of the downstream PI3K/Akt pathway associated with TCR signals to manifest negative T cell regulation by PD-1 [51]. Following this, a known ligand for the CTLA-4 inhibitory immune checkpoint molecule, CD80, was discovered to be a second binding partner for PD-L1 [52], where a specific interaction between PD-L1 and CD80 was found to have a dissociation constant (Kd value) of 1.9 μM that is three times weaker than the interaction with PD-1, with Kd value of 0.59 μM. Nevertheless, this dictates that PD-L1 could impart an inhibitory effect on T cells, even when they lack PD-1 [53]. A very recent report has demonstrated the expression of PD-L1 on disease-infiltrating T cells, where they transduce bidirectional signals (a) forward signaling and (b) backward signaling, which brings converged function to suppress the immune cell networks. During forward signaling modus, PD-L1 binds to the PD-1 receptor expressed on macrophages and reprograms the macrophages into immune-suppressive type (M2) to promote immune regulatory milieu. Concurrent to this, when PD-L1 binds to PD-1 expressed on cytotoxic T cells, they tend to downregulate the effector molecules of T cells, such as TNF-α, Interferon-gamma (IFN-γ) and granzyme B. During backward signaling modus, the T cell-expressed PD-L1 signals back to the T cell itself, by binding to its counter receptor PD-1, where they tend to repolarize the CD4^+^ from TH_1_ cells to TH_17_ cells by down-modulating the transcription factor T bet and further hampering the CD8^+^ T cells to gain effector functions [54]. In addition, there is an interaction between PD-L1 to PD-1 in cis—i.e., these molecules bind with each other that are expressed on the same cell, signifying a high likelihood of unleashing many unbound molecules—either PD-L1 or PD-1 in trans, which are expressed by other cells [55]. Despite cell membrane-bound forms, these molecules also exist in soluble (s) forms that circulate in human serum or plasma in both normal and diseased conditions [56,57]. sPD-1 and sPD-L1 are produced either due to the proteolytic cleavage from cell membrane [58] or due to the translation of alternative spliced mRNA variants, lacking the transmembrane domain [59,60]. Studies have, in part, demonstrated that sPD-1 dramatically binds to cell membrane-bound PD-L1 or PD-L2 and could functionally block the normal interaction between membrane-bound PD-1 and PD-L1 and PD-L2 to counteract the PD-1-mediated regulatory effects on T cells [60,61].

## 3. Relevance of PD-L1/PD-1 Axis on Vascular Endothelium and Barrier Functions

Vascular endothelial cells (VECs) cover the innermost wall of arteries, capillaries and veins, with approximate dimensions of 30–50 μm in length, 10–30 μm in width and 0.1–10 μm in thickness. Since vascular endothelial cells are the main sentinels of the vessel wall which play a decisive role in preserving the barrier function between circulating blood components and the underlying cellular compositions and structure of a blood-transporting vessels, they contribute to the tight regulation of: (i) balanced vasoconstriction and vasodilation; (ii) movements of the blood fluidic fraction, electrolytes, solutes and macromolecules across the blood vessels; (iii) permeability to plasma lipoproteins and (iv) platelet aggregation and leukocyte adhesion and transmigration, under steady-state conditions, and thereby contribute to vascular homeostasis [62,63]. However, altered hemodynamics and inflammatory stimuli breach the barrier to provoke vascular endothelial dysfunction that is strongly linked to atherogenesis [64,65,66]. Though the PD-1/PD-L1 pathway has been greatly investigated in various organ-associated endothelial cells [67,68,69], studies on this pathway with regard to the vascular endothelium relating to atherosclerosis are limited. Further, the pattern of PD-L1 expressions on these endothelial cells largely varies depending on the localized organs, cell activation status and the species involved, as summarized in Table 2. Interestingly, some authors speculate that the distribution of PD-L1 on the vascular endothelium might even differ between arterial and venous vessels, where explicit research documentations are still lacking to date [70]. One possible reason might be due to the 70-fold lower pressure and shear stress, experienced by veins when compared to the arterial vessel wall [71]. While an excessive shear stress leads to an endothelial injury, an increased shear stress usually generates nitric oxide (NO) from L-arginine, via arginine metabolism, in the presence of nitric oxide synthase (eNOS) cofactor, tetrahydrobiopterin (BH_4_), the process that is vital for vasodilation, where BH_4_ might inhibit PD-L1 expression [72] and, henceforth, might account for dissimilar PD-L1 expression. Furthermore, this concept might also underscore the differences in PD-L1 expression during vasoconstriction and vasodilation, which requires further investigations. Human umbilical vein endothelial cells (HUVECs) do not exhibit a constitutive expression of PD-L1 [60] under steady-state conditions, but are rather upregulated when treated with IFN-γ and TNF-α, which is in contrast to the PD-L2 distribution pattern [73], indicating that key pro-inflammatory cytokines, IFN-α, β and γ, and TNF-α, are the main drivers for enhanced PD-L1 expression on endothelial cells. Besides these cytokines, other stimuli such as vascular endothelial growth factors (VEGFs) are also involved in regulating the expression of PD-L1 on VECs [74].

During inflamed conditions and with sufficient IFN-γ, PD-L1 molecules are induced on blood vessel Endothelial Cells (ECs) to promote crosstalk with infiltrating PD-1-expressing T cells. The forward signals transmitted by PD-L1 to the PD-1^high^ T cells downregulates the T cell activation which results in either apoptosis or suppression of CD4^+^ and CD8^+^ T cell proliferation [75,76]. This, however, requires cell to cell interactions—i.e., a firm contact between the ECs and the T cells is involved, where a PD-L1 blockade, but not PD-L2, could revert the effector function responses of CD8^+^ T cells, by secreting IFN-γ [63], in response to the antigens presented by the endothelium, which could be regarded as an early step in the pathogenesis of atherosclerosis [77,78]. Since PD-L1 is a critical immune-inhibitory checkpoint for maintaining peripheral immune tolerance, the PD-L1 deficiency meant the ECs succumbed to CD8^+^ T cell-mediated attacks, where an EC is killed by CD8^+^ T cells via perforin-mediated cytolysis [79]. This aggravated cytolytic action of CD8^+^ T cells further compromises the vascular integrity, leading to the leakage of the injured vessels [79], indicating the vitality of PD-L1 expression on VECs to reduce the vascular insult. In line with this, notch signaling, achieved by the interaction between Notch and Jagged, is considered to be the homeostatic regulator of the endothelium, by enabling ECs to (i) resist apoptosis; (ii) arrest cell cycle and to promote ECs’ life span; (iii) build-up the functional blood vessels from a damaged vessel [80,81]. Intriguingly, notch signaling positively regulates the expression of PD-1 and anti-inflammatory cytokine, IL-10, on LPS-induced APCs such as monocytic cell lines [82]. A similar phenomenon was noticed on PD-L1^high^ cells that equally tend to overexpress the notch 3 molecules, where down-modulation of notch 3 proteins hampered PD-L1 expression [83], indicating a strong link between optimal levels of notch signaling and PD-L1 expression.

Although heightened expression of PD-L1 attenuates T cell-mediated cytolytic insults on ECs, the natural barrier functions of ECs are indeed disrupted by PD-L1^high^ ECs, where PD-L1 is thought to contribute to the downstream reprogramming of EC activation, affecting the vascular integrity [89]. One of the major endothelial cell-specific tight junctional proteins is an adhesion molecule, such as VE cadherin (CD144), which is indispensable for maintaining the inter-ECs’ cell contact stability and the molecular permeability of high-molecular weight plasma components and, therefore, it controls vascular integrity [97]. In addition to this, Zonula occludens-1 (ZO-1) is another 220-kDa protein of the membrane-associated guanylate kinase; its expression and distribution are pivotal for the ECs’ tight junction formations. Indeed, the expression of ZO-1 is highly regulated by undisturbed VE cadherin molecules [98,99]. In line with this, a loss of VE cadherin expression and ZO-1 occurred on the ECs monolayers with PD-L1 sufficiency [89], whereas the levels of the junctional molecules were shown to be intact even with TNF-α stimulation under PD-L1-deficient conditions, implying an uprooting quality of PD-L1 on EC-associated vascular barrier integrity.

**Figure 1:**
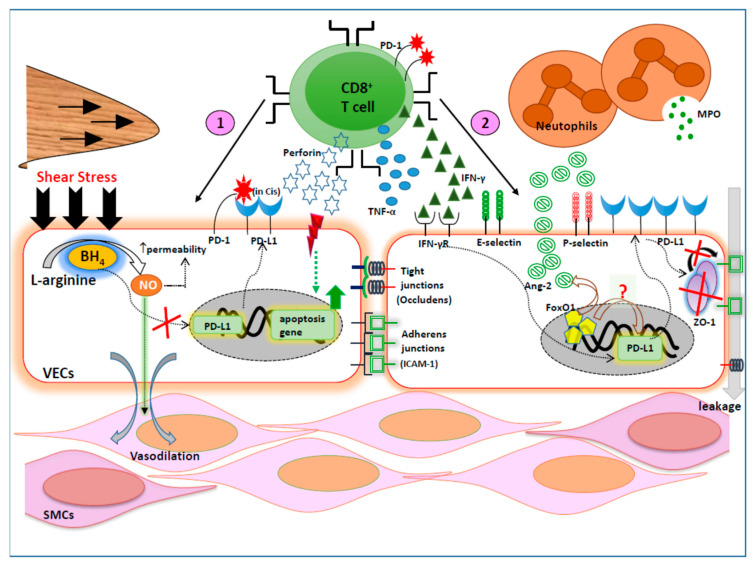
Role of PD-L1 on vascular endothelial cell injury and barrier functions. Regulation of blood vessel inflammation by PD-L1/PD-1 axis. An increased shear stress causes vascular endothelial cells (VECs) to produce nitric oxide (NO) from L-arginine in the presences of one of the several nitric oxide synthase (eNOS) cofactors, such as tetrahydrobiopterin (BH4). The generation of NO leads to vasodilation of smooth muscle cells (SMCs) and increases the VECs’ permeability. The cofactor, BH4, prohibits the PD-L1 gene transcription and reduces the expression of PD-L1 proteins on VECs. (1) Minimally expressed PD-L1 molecules have a higher possibility of binding to PD-1 molecules in cis, expressed on VECs. Diminished expression of PD-L1 boost the aggressiveness of pro-atherogenic CD8^+^ T cells, leading to VEC injury via cytolytic enzymes such as perforin and pro-inflammatory cytokines, TNF-α and IFN-γ. This insult to the VECs results in the expression of pro-apoptotic genes and enhances VEC apoptosis. (2) The released IFN-γ from CD8^+^ T cells binds to IFN-γ receptors and further induces PD-L1 protein expression on VEC surfaces. Increased PD-L1 hampers zonula occludens-1 (ZO-1), which regulates the expression of tight junctional molecules. PD-L1-mediated ZO-1 dysregulation shatters the junctional proteins, which breaches the VEC barrier causing leakage. The enhanced PD-L1 molecules also increased the angiopoietin (ang-2), an inflammatory marker, that act via its receptor Tie 2 and are tightly controlled by foxO1 transcription factor. However, the potential link between foxO1 and PD-L1 remains obscure. The cell-adhesion molecules, ICAM-1 and VCAM, are upregulated by VECs under the influence of ang-2. In turn, VEC-released ang-2 recruits neutrophils to the site, where neutrophils are sensitized to produce myeloperoxidase (MPO) and results in severe inflammation.

Next, angiotensin 2 (ang2), a pro-inflammatory molecule of the angiopoietin-Tie receptor system, has been found to express on ECs [100,101]. The levels of ang2 are low in the stable and quiescent ECs from a mature vessel but are strongly elevated during inflamed conditions with a TNF-α stimulus [101]. The ang2 binds to its receptor, Tie2, which is chiefly expressed in ECs [101]. Here, ang2 acts as a natural antagonist for the Tie2 receptor [102] by decreasing the phosphorylation of Tie2 and competitively inhibiting the binding of angiopoietin-1 (ang-1) to Tie2, thereby disrupting the protective signaling mediated by ang-1 in vascular stability induction [103]. The ang2 released from ECs promotes (i) the infiltration of neutrophils and their activity, as measured by myeloperoxidase (MPO), and with enhanced neutrophil adherent capability to the vessels [104]; (ii) change in the EC phenotype by elevated expression of P-selectins [105] and E-selectins, VCAM and ICAM-1 [106]; (iii) vessel destabilization [100]. Of note, the secretion of ang2 is under the control and tight regulation of the transcription factor FoxO1 [107]. Though the nuclear accumulation of FoxO1 is needed for the sustained expression of PD-1 and for the survival of PD-1^high^ CD8^+^-exhausted T cells [108], the relationship between FoxO1 and PD-L1 remains obscure and is not yet reported. Surprisingly, the endothelial cell release of ang2 was significantly reduced in thrombin and TNF-α-stimulated PD-L1^-/-^ EC monolayers [89], signifying a novel and indirect link of the endothelial cell regulation of PD-L1 by ang2 at the gene level, which requires further investigations. This could be well corroborated with another cell type, where excessive serum-circulating ang2 fosters the upregulation of PD-L1 on M2-type macrophages [109]. Further, the interaction between blood-circulating neutrophils and ECs decreased in PD-L1-insufficient mice. On the other hand, angiotensin II (AII), an effector molecule of the angiotensin-renin system [110], was also reported to induce endothelial expression of ang-2, mainly through one of its receptors, angiotensin receptor 1 (ATR1), which is expressed on endothelial cells [111]. Of note, the interaction between AII and ATR1 was shown to induce the upregulation of PD-L1 and further increase the stability of PD-L1 transcripts by an RNA-binding protein, human antigen R [112]. This evidence further indirectly supports the involvement of ang-2 in the upregulating endothelial PD-L1 molecule, which is also probably in association with AII. Taken together, PD-L1 contributes to EC activation, neutrophil recruitment and loss of vascular barrier integrity, by coordinating with ang2 [89]. Due to these multi-faceted features of PD-L1 on the vascular endothelium, a careful measure must be taken to manipulate PD-L1 for therapeutic immune modulatory purposes on atherosclerosis-associated blood vessel inflammatory diseases. The possible roles of PD-L1 on the vascular endothelium are depicted in Figure 1.

## 4. Regulation of Blood Vessel Inflammation by PD-L1/PD-1 Axis

Studies that detail the role of the PD-1 and PD-L1 molecules in experimental animal models of atherosclerosis as well as in human specimens with underlying atherosclerotic blood vessel inflammatory diseases are very limited.

### 4.1. PD-1/PD-L1 Axis in Experimental Atherosclerosis

Accumulating evidence has disclosed the role of the PD-1/PD-L1 pathway in downregulating proatherogenic T cell responses and upregulating the antiatherogenic effects in the atherogenic model [113,114], where an ablation of low-density lipoprotein receptor (LDLR^-/-^) genes resulted in hypercholesteremia and led to atherosclerosis. The macrophages and DCs isolated from aortic lesions of these mice expressed sufficient levels of PD-L1 molecules. When the LDLR-deficient mouse was also knocked out for both PD-L1 and PD-L2 genes, it led to: (i) an infiltration of increased numbers of lesional CD4^+^ and CD8^+^ T cells and macrophages; (ii) a burden of atherosclerosis throughout the aorta; (iii) elevated serum levels of TNF-α; (iv) highly potent APCs as T cell stimulators [115], indicating dysregulated systemic T cell responses for the development of arterial diseases [116]. Likewise, PD-1 gene deletion in LDLR-deficient mice revealed more atherosclerotic lesions in the entire aorta distal to the aortic root with an abundant infiltration of CD4^+^ and CD8^+^ T cells and macrophages. This is associated with the increased apoptosis of smooth muscle cells (SMCs) and endothelial cells in the lesion that was mediated mainly by CD8^+^ with increased cytotoxicity. In the extra lesional areas, the effector/memory CD4^+^ and CD8^+^ T cells were also increasingly distributed in the blood, spleen and peripheral lymph nodes [117]. Further RNA studies have revealed that the T cells from these severely inflamed mice showed an elevated expression of pro-inflammatory cytokine genes—*ifnγ* and *tnf*—and chemokine receptor genes—*ccr5*, *ccr6* and *cxcr3*—indicating the competence of T cell migration to the site of vessel wall inflammation. These data clearly emphasize the significance of PD-1 molecules for limiting pro-atherogenic immune responses in experimental atherosclerosis models [118]. While IFN-γ-secreting T cells promote atherogenesis, Foxp3^+^ regulatory T cells (Tregs) inhibit the formation of blood vessel lesions by attenuation of innate and adaptive responses [119,120]. In line with this, mice with PD-1 and LDLR gene deficiencies indeed provoked the systemic expansion of atheroprotective CD4^+^CD25^high^ FoxP3^+^ Tregs in blood and spleen. This expansion was noticed in parallel to the amplification of proatherogenic CD4^+^ T cells with high amounts of IFN-γ being produced; Th_17_ polarization occurred to a lesser extent. The high content of these Tregs was evident in atherosclerotic vessels, including the aortic root and the aorta, and, to our surprise, PD-1 shortages did not affect the suppressive function of Tregs. Despite Tregs expansion, an exacerbated atherosclerotic lesion and dyslipidemia persisted and advanced towards atherosclerosis due to a proportional increase in lesional T cells—macrophages that were lacking with PD-1 expression [117]. Besides, mice fed with a high-cholesterol diet, which are also prone to developing atherosclerosis, induced the expression of PD-L1 on splenic marginal zone B’s (MZB) cell surface. MZB expressed PD-L1 and suppressed the motility, differentiation and proatherogenic responses mediated by follicular helper T cells, showing the extensive role of the PD-L1/PD-1 axis in hampering proatherogenic responses [121].

### 4.2. PD-1/PD-L1 Axis in Human Atherosclerosis

Several studies have reported the frequencies of pro-atherogenic and antiatherogenic immune cells expressing PD-1 and PD-L1 molecules and their functional consequences on the blood and/or arterial tissue specimens obtained from patients with underlying atherosclerosis [122,123]. The expression of PD-1 proteins was seen on subsets of the CD4^+^ T cell phenotype, CD4^+^CXCR5^+^ T cells, in CAD patients [122] and was found elevated on CD8^+^ T cells isolated from plaques of femoral arteries and peripheral blood in patients with atherosclerosis [124]. In fact, the threshold for the expression levels of PD-1 is mainly defined by PD-L1 accumulating on the surface of APCs, such as macrophages [29]. The peripheral blood monocyte-derived macrophages from CAD patients failed to support the activation of bystander CD4^+^ T cells, reflected by decreased CD25 and CD69 markers, and cell cycle rate. Later, it was noticed that these CAD-specific macrophages had an immune-inhibitory phenotype that displayed a higher surface density of PD-L1 expression. An analysis of different regions of lesional atherosclerotic sections (early to advanced type), including fatty streak lesions, fully developed atheromas and fibrocalcified lesions, consistently found higher levels of PD-L1 on CD68^+^ macrophages. When this PD-L1 pathway was curbed during an in vitro-based interaction between CD4^+^ T cells and PD-L1^high^ macrophages, the expressions of CD25 and CD69 were substantially increased in CD4^+^ T cells which was further assisted by an improved cell proliferation rate [123]. Likewise, the PD-L1 blockade has also re-invigorated IFN-γ-compromised CD8^+^ T cell responses against the varicella virus [125], which causes latent herpes infection, as approximately 90% of world population possess the latent zoster infection; reactivation of the virus occurs in almost 50% of them and could promote vasculopathy to increase the risk of stroke and cardiovascular events, even among patients above 40 years of age [126]. These findings imply that the fate of quenched CD4^+^ and CD8^+^ T cells was purely dependent on increased PD-L1 proteins [123,125]. In contrast to this, the PD-1^high^ CD8^+^ T cells showed an increased multiplication rate in atherosclerotic patients [124]. A possible reason for this could be that the transcription factor FoxO1, which tightly regulates the sustained expression of PD-1, might play a key role in provoking proliferative activity inside CD8^+^ T cells, irrespective of the PD-1 abundance on their surface [108]. Here, PD-1 upregulation was mainly restricted to the central memory-type CD8^+^ T cell subsets which acquired antiatherogenic properties by boosting the production of IL-10 cytokines and, at the same time, by halting the synthesis of pro-atherogenic cytokines—IFN-γ and TNF-α. Of note, in addition to IL-10, PD-1^high^ CD8^+^ T cells were shown to secrete IL-4 cytokines [124]. There is a possibility that IL-4-secreting CD8^+^ PD-1^+^ T cells could be involved in inducing the production of immunoglobulins from B cells [127]. Detailed investigations are required to find out whether these produced immunoglobulins are the autoantibodies that are directed towards the antigens in atheroma plaque [128]. These effects were reversed by blocking the PD-1 molecules, as evidenced by the increased levels of IFN-γ and TNF-α and decreased IL-10 levels, and this was further confirmed by corroborating with increased T-bet and decreased GATA-3 transcription factors, respectively. Thus, clonal expansion of bystander pro-atherogenic T cells could be prevented when PD-1 is abundantly expressed [124]. Contradictorily, increased PD-1 proteins on the T cell subset that lacks CD28 molecules (popularly known as CD4^+^ CD28^null^ T cells), which destabilizes the plaques with their in-built ability to release atheroma-deteriorating secretomes—IFN-γ, TNF-α, perforin and granzyme B—were increased among patients with aggressive atherosclerosis macrovascular disease with underlying type 2 diabetes mellitus (T2DM) comorbidity [56]. However, sera from these patients were noticed for accumulated sPD-L1 that positively correlated with IFN-γ levels. This raises the possibility of blocking the membrane-bound PD-1 and PD-L1 pathway by sPD-L1 in atherosclerotic patients and thereby renders the severity of PD-1^+^CD4^+^CD28^null^ T cells to amplify and worsen the intensity of ongoing inflammation, causing vessel wall damage, and, finally, culminating in atherosclerotic plaque rupture [129]. A differential pattern of PD-1 expression was noticed between CD4^+^CD45RO^+^ (memory-type) and CD4^+^CD45RO^−^ (effector-type) T cells, with highly inclined expression in the memory T cell-type, whereas PD-1^dim^ memory T cells produced copious amounts of IFN-γ and the PD-1-sufficient memory T cells skewed towards TH_17_ polarization and produced IL-17 cytokines [130]. This difference could warn us to carefully evaluate the expression pattern of PD-1 and its role in different types of polarized T cells in atherosclerotic blood vessel diseases.

Interestingly, several potential self-antigens were described in context with CAD [131] and in line with this, keratin 8 was recently shown as a self-antigen to elicit effector CD4^+^ and CD8^+^ T cell responses in CAD patients [132]. As expected, the stimulation of CAD PBMCs with keratin 8 did not tend to elevate the PD-1 transcripts, signifying an aberrant peripheral tolerance in CAD patients due to massive inflammatory responses against the self-antigen which could be the cause for impaired PD-1 expression, while keratin 8 increased the PD-1 mRNA on PBMCs obtained from control subjects [132]. This shows that PD-1 is induced by self-antigens to restrict the T cell reactivity to the underlying tissues under normal circumstances, whereas this phenomenon fails among CAD patients. Consistent with this, a report has shown inadequate levels of PD-1 on T cells and PD-L1 on myeloid (m) DCs in the peripheral blood of CAD patients, demonstrating a strong stimulant nature of CAD mDCs to activate T cells that are further accompanied with IFN-γ and IL-2 cytokine production. However, this minimally expressed PD-L1 could be reversed back to a normalized level with IFN-α treatment on CAD-derived mDCs, which was assisted by decreased IL-12 and increased IL-10 cytokines. When these IFN-α-upregulated PD-L1 molecules were triggered, the proliferation and IFN-γ and IL-2 cytokine production of cocultured allogeneic T cells declined. This is a typical negative feedback loop mechanism, where inflamed T cells shooting with IFN-γ/α tend to bring autocrine and paracrine effects on self and neighboring cells to upregulate PD-L1 via the IFN-dependent system [133]. The key question is whether such a negative feedback loop mechanism works in patients with atherosclerotic blood vessel diseases, as inflammation exerts dominant effects on immune networks and the disease progress.

Next, a perturbed regulatory microenvironment was noticed in CAD patients that was reflected by lowered IL-10 cytokine secretion, where a decreased blood frequency of regulatory T cell type 1 (Tr1), characterized by cell surface molecules, CD4^+^CD49^+^LAG3^+^CD45RO^+^ T cells, was evident [134]. These CAD-specific Tr1 cells were incapable of mediating the suppressive effects on conventional T cells, mainly through IL-10-dependent manner. The frequencies of Tr1 cells, LAG3 expression and IL-10 production were negatively correlated with the body mass index (BMI) of CAD patients, reconfirming the cardioprotective attribute of Tr1. Here, CAD-specific Tr1 exhibited increased levels of PD-1 molecules but with decreased levels of CTLA-4 on its surface, indicating a disunion between PD-1 upregulation and IL-10 production. This was confirmed by other reports showing negligible effects of the PD-1 blockade on IL-10 cytokine secretion [135,136]. In contrast, natural Tregs expressing CTLA-4, GITR along with high levels of PD-1 tend to release IL-10 and TGF-ß when bound to the PD-L1 expressed by oxidized (ox)-low-density lipoprotein (LDL)-stimulated HUVECs [95], summarizing a possibility for an alternative mechanism by which EC–Treg interactions induce the suppression of inflammation in atherosclerosis. Nevertheless, the vitality of the PD-1/PD-L1 axis from the aforementioned in-vitro studies became more evident and valid when the malignant patients (>80%) were treated with the PD-L1 blockade, leading to severe-to-fatal cardiovascular immune-related adverse events [137]. Thus, clinical phase trials targeting other diseases further reinforce the concept of aggravating the atherosclerotic blood vessel disease when the PD-L1/PD-1 axis is being hampered.

## 5. Delicate Restoration of Protective Immunity to Curtail Infections by Targeting PD-1 Pathway in Underlying Blood Vessel Inflammation

Though alteration in numbers, modified dynamics and spatiotemporal distributions arise with innate and adaptive immune cells, where inflammation-seeking monocytes recruit T cells by adhering to the VECs and cause endothelial dysfunction during blood vessel inflammation [138], the virulence of invading pathogens provokes additive effects in exacerbating the immune responses and worsening the existing state of underlying chronic inflammation [139,140]. Though T cells are the predominant immune cell type in human atherosclerotic lesions, as assessed by single-cell RNA sequencing and mass cytometry [141], the defective responsiveness in T cell-mediated immunity to conquer infections persists. One of the main reasons behind dysfunctional protective T cells is the expression of PD-1 and PD-L1 or PD-L2 molecules.

### 5.1. PD-1/PD-L1 Axis during Acute Infections in Blood Vessel Inflammation

Acute infections, such as influenza and coronavirus (SARS-CoV-2) pose a significant risk of mortality in patients with underlying chronic blood vessel inflammatory diseases [142,143]. When infected in atherosclerosis-prone apolipoprotein E-deficient (ApoE^−/−^) mice, the influenza virus imparts its pathological imprint on the vascular system in multiple ways, resulting in (i) massive infiltration of inflammatory cells, macrophages and T cells; (ii) proliferation of smooth muscle cells; (iii) fibrin deposition and increased procoagulant effects; (iv) platelet aggregation and thrombosis; (v) increased amounts of pro-inflammatory and prothrombotic cytokines; (vi) endothelial dysfunction; (vii) release of endogenous catecholamine [139,144]. Following influenza infection, the expression of PD-L1 is strongly induced in mouse lung epithelial cells in an IFN-α receptor (IFNAR)-dependent manner that rapidly infiltrated the PD-1^high^CD8^+^ effector T cells to the infected airways. When the PD-L1 is blocked, the functionality of CD8^+^ T cells was improved in terms of the production of IFN-γ, granzyme B and upregulation of the degranulation marker, CD107ab. This led to an accelerated viral clearance and recovery of disease course. However, infection with high pathological influenza strain, ΔVn1203, which is a recombinant virus with the six internal PR8 genes and surface H5N1 proteins from A/Vietnam/1203/04, similarly induced PD-1^high^-expressing CD8^+^ T cells that compromised the protective immunity against influenza clearance, but the in vivo blockade of PD-L1 expression did not rejuvenate or completely rescue the polyfunctionality of the T cells, though the numbers of CD8^+^ T cells were increased with fairly reduced viral tires [145]. This implies that the therapeutic effectiveness of disrupting the PD-1/PD-L1 pathway during the course of acute influenza infection depends on the type of invading influenza serotypes. The murine studies could partly be corroborated by human studies, where an increased expression of PD-L1 on DCs and CD4^+^ and CD8^+^ T cells and restricted PD-1 levels on T cells were reported to be more evident with the highly pathogenic A (H1N1) pdm09 pandemic strain rather than the mild H3N2 seasonal influenza strain. The expression of PD-L1 lowered T cell proportions, promoted CD8^+^ cell apoptosis, and decreased the production of IFN-γ, TNF and IL-10 cytokines [146]. Though there is no direct report indicating the triad relation between the expression of PD-1 or PD-L1, influenza virus and cardiovascular disease, exclusively on CAD, PAOD, CS, there are several studies dictating the possible role of influenza infection in accelerating mortality among the patients with underlying cardiovascular diseases [142,147]. While the expression of a high level of PD-1 on CD8^+^ T cells impede the cytotoxicity to influenza-infected cells [146], the expression of PD-1 on follicular helper T cells (T_FH_ cells) is indeed required for an efficient production of influenza-specific antibodies in the germinal centers, although the precise function of PD-1^high^-expressing T_FH_ cells is obscure [148,149]. This underscores that a generalized targeting strategy to block the PD-1/PD-L1 pathway could still remain as a major drawback due to the differences in the expression and functionality of PD-1 on various immune cell types.

In addition to this, patients with cardiovascular comorbidities (e.g., CAD) acquire a heightened and unique risk of accelerated mortality in critical care units during SARS-CoV-2 viral infections, which causes the disease COVID-19 [150], a current serious global threat, which the world health organization has declared as public health emergency of international concern and then a pandemic in March 2020 [151]. The state “lymphopenia” existed among both younger and older patient categories with COVID-19 infection, where blood-circulating CD4^+^ and CD8^+^ T cells were drastically reduced in numbers. This was accompanied by an increased expression of PD-1 molecules and apoptotic marker, CD95, on T cells and was ascertained regardless of age. The PD-1 levels were positively correlated with CD95 on T cells, indicating the bias of T cells towards exhaustion and apoptosis [152]. This clearly shows that the SARS-CoV-2 virus is intensifying PD-1 protein expression on T cells and driving them to a state of exhaustion and hampering the protective T cell immunity, especially among those who are in a critical condition requiring intensive care unit (ICU) support. Of note, PD-1^high^ T cells were increased from preclusive to advanced symptomatic stages of COVID-19 [153]. Furthermore, higher serum circulating levels of IL-10 and IL-6 cytokines was highlighted as one of the prime mechanisms behind the cause of COVID-19 severity, by negatively regulating protective T cell survival and proliferation [153]. Intriguingly and conflicting to this, a higher degree of T cell proliferation, expression of activation marker, HLA-DR, and production of cytolytic enzymes, perforin and granzyme B, were noticed, despite a high expression of PD-1, among severe infection groups when compared to mild COVID infection groups. The obvious reason was considered to be the aberrant T cell hyper-reactivity to lung parenchyma causing excessive damage during COVID-19 severity [154], though the exact functional role of PD-1^high^ CD4^+^ and PD-1^high^ CD8^+^ T cells have not been commented on. Although a bidirectional interaction between COVID-19 infection and a series of cardiovascular events was evident, the SARS-CoV-2 virus-induced systemic inflammation was suspected to accelerate the de novo cause for cardiovascular damage. However, the exact mechanism underlying such interactions remains elusive and would remain interesting to explore such interactions together with the pattern and kinetics of PD-1 expression on T cells and PD-L1 on APCs [155].

### 5.2. PD-1/PD-L1 Axis during Chronic Infections in Blood Vessel Inflammation

One interesting reason behind the genesis of atherosclerosis, an underlying pathophysiological cause for chronic blood vessel inflammatory diseases, was reported to be an inherent quality of certain pre-existing pathogens, including cytomegalovirus, chlamydia and helicobacter species that causes latent, chronic infections in the human community [156]. The possible mechanisms of chronic infection-induced atherogenesis could involve endothelial cell injury, increased expression of various adhesion molecules and inflammatory cytokines, macrophage-activated plaque destabilization, localized hypercoagulability, and molecular mimicry. Most importantly, the persistence of PD-1^high^ CD8^+^-exhausted T cells is the hallmark behind chronic activation, due to constant exposure to the intrinsic antigenic components [157].

#### 5.2.1. Role of PD-1/PD-L1 Axis in Cytomegalovirus-Associated Atherosclerosis

In 1987, Adam et al. were the first to report the potential link between the chronic cytomegalovirus (CMV) infection and the pathophysiology of atherosclerosis [158]. Serological and molecular examinations have revealed the increased circulating titers of CMV-specific antibodies among CAD patients and the presence of CMV gene fragments in patients with bilateral carotid artery stenosis [159,160]. In fact, human CMV establishes the latency by utilizing the endothelial cells as one of the cellular reservoirs and initiates the coagulation cascades, assisted with the abundant levels of von Willebrand factor (vWF) and cell-adhesion molecules such as ICAM-1 and VCAM-1 that further aid in platelet aggregation and thrombus formation [161]. Such a dynamic process of acute thrombosis formation in the coronary arteries leads to MI [162]. Since cytotoxic CD8^+^ cells readily upregulate PD-1 during chronic activation which dampens the effector responses, the loss of CMV-specific CD8^+^ T_EMRA_ (effector memory RA^+^) cells occur among CMV-positive patients with underlying MI. This loss is mainly due to spontaneous apoptosis manifested on CD8^+^ T_EMRA_, expressing higher levels of PD-1 molecules, which defines an inability to control the latent infection. Here, PD-1^dim^- and PD-1^high^-expressing CD8^+^ T cells displayed increased CD8^+^ T cell death compared to PD-1^neg^ cells. This explains the vulnerability to CMV-induced MI in CMV-positive patients, assisted by the vital role of PD-1 in CD8^+^ T cell depletion [163].

#### 5.2.2. Role of PD-1/PD-L1 Axis in Chlamydia-Associated Atherosclerosis

*Chlamydia pneumoniae (C. pneumoniae)* is a respiratory pathogen that has been recognized as a possible etiology for the inflammatory activities of atherosclerosis, where the antibodies against chlamydia antigens were strongly associated with the development of coronary heart disease [164]. *C. pneumoniae* has been isolated from coronary [164], carotid [165] and peripheral arteries [166], as it exhibits tropism for atherosclerotic lesions and is therefore found in higher titers at the lesion. This pathogen can invade and persist in several cell types at both respiratory and cardiovascular sites, including circulating monocytes, DCs, macrophages, aortic smooth muscle cells, and vascular endothelium, where infected circulating monocytes transmigrate into the vessels and interact with the vascular endothelium by cell–cell contact to trigger a series of inflammatory reactions leading to the release of pro-inflammatory cytokines and procoagulants and recruitment of chlamydia-specific T cells to initiate atheroma formation [167,168,169]. Although T cells, plasmacytoid DCs and monocytes represent the major sources for PD-1 and PD-L1 expression during the respiratory chlamydia infection that leads to an airway hyperresponsiveness (AHR) [170], there is no report that documents the role of the PD-1/PD-L1 axis in chlamydia-associated atherosclerosis. With underlying AHR, it has been stated that the early-life infection with respiratory chlamydia had expressed PD-L1 on leukocytes that led to the secretion of T_H2_ (especially IL-13) cytokines, which aggravated AHR during adulthood. Since there exists a disparity between IL-13 cytokines exhibiting either an atheroprotective function via enhanced M2 macrophage polarization [171] or a pro-atherogenic function by increasing CD36 signaling required for the macrophage foam cell formation [172,173], a thorough investigation on PD-L1-induced IL-13 in chlamydia-induced atherogenesis is highly recommended.

#### 5.2.3. Role of PD-1/PD-L1 Axis in Helicobacter-Associated Atherosclerosis

*Helicobacter pylori* is a Gram-negative, spiral extracellular bacterium that infects the gastric mucosa and thereby causes various types of gastrointestinal diseases, including peptic ulcers, chronic gastritis, and gastric cancer [174]. Though detailed mechanisms remain obscure, numerous studies have shown a direct relation between cytotoxic-associated gene-A (Cag-A) positive *H. pylori* strains and blood vessel diseases, such as CAD [175], MI [176], PAOD [177] and stroke [178], merely by demonstrating (i) a higher IgG seropositivity, (ii) an increased thickness of carotid plaque and enhanced plaque vulnerability [179], (iii) an increased carotid pulse wave velocity [180], and (iv) modified ox-LDL levels and high sensitive C-reactive protein (hsCRP) levels [181]. Furthermore, *H. pylori* infection was associated with the modified atherogenic lipid profiles, including increased serum triglyceride, total cholesterol concentrations and decreased HDL cholesterol concentrations [182]. The helicobacter-infected gastric epithelium tends to express higher levels of surface PD-L1 proteins, either upon direct *H. pylori* cell contact or by indirect secretory virulent factors, cag-A, urease B, leading to (i) the suppression of CD8^+^ and CD4^+^ T cell proliferation, (ii) reduced IL-2 cytokine release and CD69 activation marker [183], (iii) apoptosis of recruited T cells [184], and (iv) induction of peripherally derived CD4^+^ CD25^+^ FoxP3^+^ regulatory T cells that further controls cytotoxic T cell proliferation [185]. Such a maladaptive immune response favors the bacterium to survive and is therefore responsible for the persistent state of chronic inflammation, which might contribute to the development of inflammation in blood vessels.

Taken together, there is almost no prominent study on the triad relationship between chronic infections such as chlamydia or helicobacter in patients with atherosclerosis, which are analyzed together with PD-1 or PD-L1 molecules. This could be an interesting subject for further investigations. Nevertheless, under such pre-existing chronic infections, it remains essential to preserve an optimal proportion of protective CD8^+^ T cells with cytotoxic potentials. Though terminal-exhausted CD8^+^ T cells express high levels of PD-1 during chronic infections, there is a possibility of re-invigorating the PD-1^high^ CD8^+^ T cells into an effector phase from a terminal differentiation state through FoxO1 that is accumulated in the nucleus [108]. Very recently, it was also shown that FoxO1 gene polymorphisms have been associated with the development of carotid atherosclerosis and were reported to be downregulated in coronary heart disease patients [186]. Not only this, FoxO1 might also function as a critical component for protecting PD-1^high^ CD8^+^ T cells from apoptosis, by increasing their survival through Bcl_2_ upregulation. This pinpoints the idea behind re-energizing an exhausted PD-1^high^ CD8^+^ T cell into an effector population, by careful examination for an optimal expression of FoxO1 in patients with atherosclerotic blood vessel diseases.

## 6. Implication of PD-1/PD-L1 Axis in the Altered Metabolism during Blood Vessel Inflammation

A cluster of health-related conditions occurs due to altered metabolisms, including modified lipid profiles with decreased high-density lipoproteins (HDLs) and increased very low-density lipoproteins (VLDLs), increased triglycerides as well as blood glucose levels and high blood pressure, which advances to metabolic syndromes such as obesity and type 2 diabetes and are independently associated with an increased risk for cardiovascular diseases, such as CAD and stroke [187]. These circulating blood cholesterols provoke crater-like defects and balloon-like protrusions on the surfaces of blood vessels, indicating endothelial cell damage, which leads to auto-oxidation and results in the generation of atherogenic oxidative derivatives [188]. Under steady-state conditions, the endothelial cells are equipped with lipid droplets, an intracellular hub for lipid metabolism, which protect ECs from lipotoxicity and supply fatty acids for proper mitochondrial functions [189]. Nevertheless, a severely altered lipid metabolism causes an accumulation and surplus of intracellular lipid pool leading to a dysfunction in mitochondria and an impairment in autophagy induction (waste recycling intracellular machinery evolved to monitor abnormal fluctuations in key metabolic parameters) that further reduces fatty acid oxidation [190,191]. These densely deposited free fatty acids could impose multiple detrimental effects on VECs which include: (i) impaired vasodilation; (ii) increased vasoconstriction; (iii) increased oxidative stress; (iv) enhanced endothelial permeability; (v) reduced tight junction proteins (ZO-1); (vi) activation of pro-inflammatory transcription factor NF-κB and activator protein 1 (AP-1); (vii) increased expression of adhesion molecules, VCAM and ICAM-1; (viii) increased plasma levels of TNF-α, monocyte chemoattractant protein-1 (MCP-1); (ix) diminished NO production, and (x) induction of apoptosis and necrosis [192]. The apoptotic endothelial cells further contribute to atherothrombosis and aggravate inflammation in blood vessels [193]. In line with these, a new mechanistic property of PD-1-expressing T cells was revealed, where PD-1^+^ T cells were shown to enhance the process of lipolysis which was determined by increased levels of the enzyme, adiposite triglyceride lipase (ATGL). They indeed promoted the ß-oxidation of fatty acids (FAO) of endogenous accumulated lipids via the rate-limiting enzyme of FAO, carnitine palmitoyl transferase (CPT1A), to ensure T cell survival and longevity [194]. On the other hand, PD-L1-sufficient cells outcompeted the infiltrating T cells in the uptake of lipids by increasing the intracellular contents of fatty acid-binding proteins (Fabp4/5), where CD8^+^ tissue-resident bystander T cells were deprived and induced to undergo cell death [195]. This mechanism could explain a rapid accumulation of lipids inside any PD-L1^high^-expressing cells. However, PD-L1-dependent regulation of lipids that are expressed on endothelial cells remains elusive and thereby requires further investigation for blood vessel inflammatory diseases, such as CAD and PAOD.

**Figure 2:**
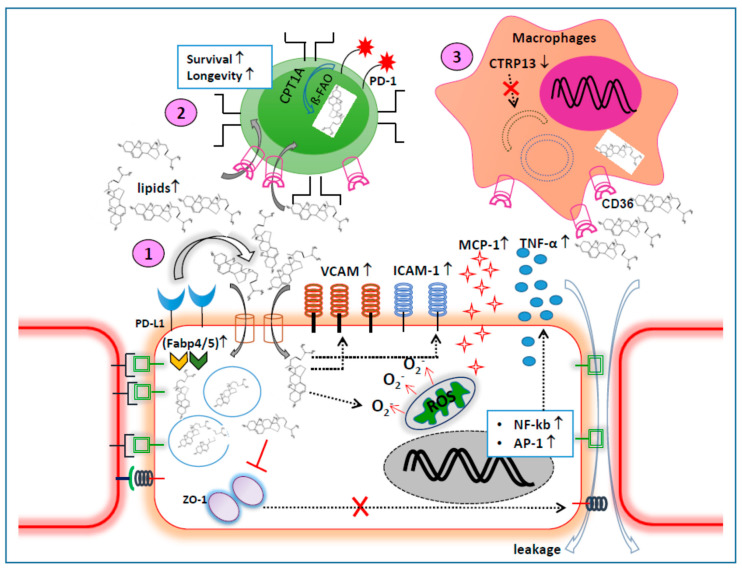
Possible mechanistic link between PD-1 and PD-L1 molecules and an altered lipid metabolism on different types of cells during vascular inflammation. Possibly, (1) an increased uptake and intracellular accumulation of lipids in VECs might induce (i) oxidative stress to mitochondria as reflected by ROS production; (ii) cell surface expression of VCAM and ICAM-1 adhesion molecules; (ii) increased secretion of monocyte chemoattractant proteins (MCP-1); (iii) increased transcriptional factor NF-κB and activator protein-1 (AP-1) expression; (iv) increased TNF-α levels; (v) decreased VEC tight junctional protein regulator, (ZO-1); (vi) enhanced permeability and increased VEC apoptosis. The lipid uptake by VECs is indeed aided by the increased PD-L1 levels through the upregulation of fatty acid-binding proteins (Fabp4/5). (2) The circulating lipids are also uptaken by bystander PD-1highCD8^+^ T cells that promotes fatty acid oxidation via carnitine palmitoyl transferase (CPT1A), the rate-limiting enzyme of FAO, to sustain T cell survival and longevity. (3) Reduced CTRP13 in CAD macrophages prohibit the autophagy flux (AF) to clear the accumulated lipids that are taken via the scavenger receptor, CD36. This tends to load the CAD macrophage with circulating lipids to convert them into a foam cell, which is a key cell type with altered phenotype and metabolism in atherosclerotic plaques.

Further, the intracellular autophagic flux mechanism controls the deposition of abnormal intracellular lipids and is, therefore, required for the breakdown of lipid droplets to decrease the augmented intracellular triglyceride levels [196]. Of note, the formation of autophagy is defective in patients with CAD as determined by a reduction in the components that are involved in autophagosome genesis, such as LC3 [197] and ATG5 [198]. Nevertheless, CVD patient-derived primary macrophages treated with the autophagy inducer rapamycin tend to increase these components with a substantial decrease in the (i) gene expression of Apo B, involved in lipoprotein metabolism, (ii) production of LDL, and (iii) production of pro-inflammatory cytokines, IL-6 and TNF-α [199]. In addition to this, a secreted adipokine, C1q/tumor necrosis factor-related protein 13 (CTRP13) involved in improving lipid and glucose metabolism, was also reduced in the plasma of CAD patients [200]. Interestingly, CTRP13 was reported to promote autophagy flux in macrophages and boosted the autophagy-lysosome fusion-dependent degradation of CD36 molecules [200], which are scavenger receptors expressed by various cell types including monocytes, macrophages, and vascular endothelial cells, and participate in the uptake of ox-LDL, forming foam cells and trapping the foam cell by hampering their migration which cumulatively contributes to the initial critical step in atherosclerosis progression [201,202]. This underscores the fact that autophagy-assisted degradation of CD36 on macrophages fails to uptake ox-LDL and thereby restricts macrophage lipid retention. Concerning pro-atherogenic T cells, releasing either IL-17 or/and IFN-γ could also experience burdened intrinsic lipid metabolism which certainly imparts serious effects on the (a) T cell phenotypic modulation by expressing inflammatory markers, CD69, IL-2 and insulin receptors (GLUT-4) and (b) T cell functions by increasing pro-inflammatory cytokines such as TNF-α, IL-1β and IL-6, depolarization of mitochondrial membrane and activation of cytochrome C, caspases (9 and 3) to induce apoptosis [203]. T cells that coexpress CD36 and PD-1 proteins result in a higher rate of fatty acid uptake by the cells. At the same time, a kinship between PD-L1 and autophagy still remains controversial, according to a few reports [204,205]. Since a defective autophagy might fail to clear the intracellular lipid pool in CVD, manipulation of the PD-1/PD-L1 axis could be achieved to drive autophagy formation, provided the PD-1/PD-L1/autophagy axis are thoroughly examined in patients with blood vessel inflammation. The cumulative functional aspects of PD-1 and PD-L1 molecules on different cell types during altered lipid metabolism are depicted in Figure 2.

Next, the altered glucose metabolism accelerates the aggressiveness of atherosclerotic inflammation, as seen in either diabetic or prediabetic metabolic abnormalities with excessive oxidative stress [206]. Of note, both monocytes and macrophages represent the key drivers of vascular diseases, where macrophages tend to maldifferentiate into an M1-polarized hyperinflammatory phenotype upon excessive utilization of glucose in CAD patients. Further, an excess availability of glucose fuels the imbalanced production of mitochondrial reactive oxygen species (ROS) that assist in the release of copious amounts of pro-inflammatory cytokines, IL-6 and IL-1β. A detailed molecular study has revealed that a redox-sensing glycolytic enzyme, pyruvate kinase (PKM2), gets dimerized, which is influenced by abundant ROS, and thereby enables its nuclear import. This nuclear PKM2 further activates STAT-3 phosphorylation to drive the intracellular synthesis of IL-6 and IL-1β. Thus, altered glucose metabolism aggravates atherosclerotic blood vessel inflammation through the glucose-ROS-PKM2-STAT-3 pathway [207]. Not only this, the PKM2 dimers harbor the capability to activate platelet and thrombus formation [208]. Interestingly, there are two distinct mechanisms by which PKM2 is reported to regulate the firm expression of PD-L1 on immune cells such as macrophages, DCs and T cells. First, the dimerized PKM2 and hypoxia-inducible factor 1α (HiF-α) bind to two hypoxia response element (HRE) (HRE1 and HRE4) regions of the PD-L1 promoter in LPS-treated macrophages [209]. Of note, the tetramerization form of PKM2 inhibited LPS-induced PD-L1 expression on the macrophages [207]. A localized oxygen deficit is a common factor in macrophage-rich areas of advancing human carotid atherosclerotic lesions [210] and since CAD macrophages displayed increased expressions of dimerized PKM2, a similar mechanism of synergistic action between PKM2 dimers and HiF-α in triggering PD-L1 on hyperinflammatory macrophages and on inflamed vascular endothelial cells in atherosclerostic blood vessel diseases are expected. Second, the dimerized PKM2 from CAD macrophages leads to a reduced glycolytic flux and thereby accumulates several glycolytic intermediates. This maximizes the load of pyruvate transport into mitochondria, causing excessive stress by generating ROS. Mitochondrial pyruvate induces the production of BMP4 and IRF1 to upregulate the surface expression of PD-L1 [123]. Thus, an excess availability of glucose from the inflamed microenvironment and its uptake and aberrant utilization by the cells is certainly linked to the upregulation of PD-L1. However, it is not yet evident whether increased expression of PD-L1 ascertains the disease amelioration as the complexity of the microenvironmental skew to shape the macrophage and endothelial cell metabolism and its impact on atherosclerosis progression.

## 7. Therapeutic Relevance of PD-L1/PD-1 Axis in Blood Vessel Inflammation

### 7.1. Current Understanding

To date, the FDA-approved drugs that are utilized to block either PD-1 (nivolumab and pembrolizumab) or PD-L1 (atezolizumab, avelumab, and durvalumab), classified under the name “immune checkpoint inhibitors (ICI)”, have made a distinct revolutionary pathway for a successful treatment and improved survival of cancer patients [211,212]. Several clinical-based works have affirmed severe immune-related adverse outcomes in the development of atherosclerosis-assisted cardiovascular diseases during tumor therapy [213,214], where 22 meta-analysis trials have reported about 3% ischemic strokes or myocardial infarction [215]. In addition, 2.6% of patients developed atherosclerotic vascular events (AVEs) that were evident within the first 6 months of ICI treatment against non-small cell lung cancer [213], indicating the rapid progression of atherosclerosis-related cardiovascular diseases within a short duration of ICI therapy. This implies that the atherosclerotic plaques might already exist among these patients rather than the de novo plaque formations [216,217]. Of note, the survival rate of patients with AVEs was poor compared to those who did not develop AVEs. Another patient with a metastatic giant bone cell tumor was favorably treated with ICI; nonetheless, a year later the same patient developed ST segment-elevation myocardial infarctions with a rapid progression of stenosis [218], raising the possibilities that ICI can promote accelerated atherosclerosis by rupturing existing plaques. Though the T cell boosting mechanism was most commonly proven to be behind the adverse effects of ICI therapy on the cardiovascular system, a very recent report, intriguingly, has demonstrated an unexplored facet of ICI treatment on the macrophage system, where ICI (especially PD-1 inhibitors) polarized the macrophage into an M1 phenotype by decreasing KLF4 (Krüppel-like factor 4, a DNA-binding transcriptional regulator), which exerts atheroprotective effects by decreasing macrophage-associated inflammation, in terms of macrophage activation and polarization [189]. This decrement of KLF4 is associated with an increase in inflammatory micro-RNA (miR) elements—in particular, miR-34a, to enable the M1-type macrophage to produce inducible nitric oxide synthase (iNOS) and pro-inflammatory cytokines, TNF-aα and IL-1β [219].

### 7.2. Future Perspectives

A thought process to achieve a targeted and desirable atheroprotective outcome to improve the clinical status of patients with cardiovascular diseases, by manipulating the PD-1/PD-L1 axis, is greatly needed. Primarily, this need has to undergo a prudent evaluation among cardiovascular patients who have no history of cancer. Otherwise, the situation becomes highly critical to finetune the PD-1/PD-L1 axis in cardiovascular patients coexisting with tumor progression, where an attenuation of the PD-1 pathway is mainly preferred and is therefore obliged to aggravate cardiovascular events. Although preclinical studies have shown the consequences of preserving PD-1/PD-L1 signaling in attenuating the pro-atherogenic immune cells, we are still far away from spanning these ultimate goals relating to clinical trials. As previously mentioned in this review, PD-L1 expression was higher among Caucasian CAD patients, in contrast to the Asian CAD patients, implying the initiatives for a broader analysis of PD-1 and PD-L1 expression patterns among different ethnic territory residents. Moreover, a series of underlying clinical parameters, including (a) chronic infections, (b) cancer, and (c) metabolic conditions, must also be thoroughly scrutinized among cardiovascular disease patients to facilitate the combinatorial therapies together with PD-1 or PD-L1 agonist therapy. In fact, a recent report has highlighted the conversion of TH_1_ to TH_17_ cell-type when PD-L1 signals in a backward mode in T cells [54]. Evidence-based reports have deciphered the involvement of TH_17_ in cardiovascular disease [220], imposing a careful evaluation that is required while considering antiatherogenic therapy with PD-L1 agonist antibodies, especially when these PD-L1 molecules are expressed on T cells. Another vital interventional factor is that the reported CAD patients displayed higher circulating levels of sPD-1 or sPD-L1 in their blood, signifying their hindrance in subsiding the inflammatory processes that are assisted by membrane-bound PD-1/PD-L1 interactions. To overcome this issue, one possibility is that a targeted antibody-mediated neutralization of soluble circulating PD-1 and PD-L1 molecules could be achieved, which would render prompt inhibitory signaling from cell surface-expressed PD-1. Other possibilities to upregulate PD-L1 and PD-1, under atherosclerotic conditions, could be achieved by manipulating the expression of notch and FoxO1 molecules, which are either directly or indirectly linked to regulate PD-1 and PD-L1 molecules.

Beyond this, a previously published article by Weyland et al. on the involvement of PD-L1/PD-1 pathway in blood vessel inflammatory disease has mainly focused on detailing the role of PD-L1^lo^-expressing DCs in a vasculitis (for example, Giant cell arteritis (GCA)) and PD-L1^hi^-expressing macrophages in chronic inflammation disease (example, CAD). Here, the report explicitly emphasizes the importance of a PD-L1 deficit in aggravating inflammatory responses such as those in GCA, whereas PD-L1 sufficiency tends to interfere with protective adaptive immunity against viral infection, as reported in CAD [221]. However, our present review differs from the previously published review in terms of detailed explanation on the role of the PD-L1/PD-1 axis, in all those vital elements of atherosclerotic inflammatory diseases, including VECs’ barrier function and integrity, disease regulation in experimental models and in human specimens, acute versus chronic viral and bacterial infections, disease-associated lipid and glucose metabolic alterations, and current knowledge in real-time clinical settings and forthcoming perspectives.

## 8. Concluding Remarks

Though compelling evidence has shed light upon the constructive role of PD-1 and PD-L1 upregulation in atherogenic cells for endorsing possible cardioprotective outcomes, detailed prospective investigations must be unquestionably carried out to investigate whether the agonistic antibodies that stimulate PD-1 and/or PD-L1 and PD-L2 molecules could converge into a rewarding therapeutic approach to treat atherosclerosis. Taken together, in this review, we have elaborated the involvement of PD-1, PD-L1 and PD-L2 in blood vessel inflammatory diseases with underlying atherosclerosis, where a precise and deeper understandings of the inhibitory mechanisms of PD-1 in atherosclerosis will uncover and support the rationale for developing PD-1/PD-L1-based immuno-modulatory therapies for diversified blood vessel inflammatory diseases.

## Figures and Tables

**Table 1:** Differential Expression of programmed death-1 (PD-1) and its ligands in vasculitides.

Blood Vessel Diseases and References	Disease-Specific PD-1, PD-L1/PD-L2 Expression, Sample Origin (Cells/Tissues) and Sample Type (DNA/Transcript/Protein)	Inferences	Species Reported	Population Studied
GCA [29,30]	1. ↑ PD-1 (t) and ↓ PD-L1 (t) on temporal arteries; ↑ PD-1 (p) and ↓ PD-L1 (p) on vascular T cells and DCs, respectively	1. Inhibiting PD-1 pathway (i) increases vascular inflammation; (ii) aggravates maladaptive remodeling of arterial wall	human and mice (NOD. Cg-Prkdcscid Il2rgtm1Wjl/SzJ (NSG))	Caucasian /Hispanian/ African-American
	2. ↓ PD-1 (p) on blood CD4+T cells and no differences PD-L1/PD-L2 (p) on blood monocytes; ↑ PD-1 (p) and PD-L1 (p) on temporal arteries	2. Minimizing immune activation and preventing further damage the vessel wall	Human	Dutch
KD [31]	↑ T allele frequency of PD-1 gene SNP (rs41386349)	PD-1 genetic predisposition in contribution to KD	Human	Korean
BD [32,33,34]	1. ↓ PD-L1 (p) on APCs and cutaneous lesions and ↓ PD-L1 (t) in PBMCs	1. Disrupted PD-L1 contribute to the development of BD	Human	Korean
	2. DNA from blood samples lack gene polymorphisms in PD-1, PD-L1 and PD-L2 (SNP’s: PD-1 rs2227981 and rs10204525, PD-L1 rs1970000 and PD-L2 rs7854303)	2. Negligible role of PD-1 and its ligands in BD	Human	Chinese Han
GPA (fWG) [35,36,37]	1. ↑ PD-1 (p) on T cells; No PD-1 (p) on lesional T cells of renal biopsies with necrotizing & crescentic glomerulonephritis	1. ↑ PD-1^+^ T cells were positively correlated with activation state including CD28^null^ memory Th cells as well as T effector memory cells, IFN-γ+ T cells, induction of PD-1 by chronic CMV infection; PD-1^+^CD4^+^ CD25^+^ T cells were negatively correlated with the relapse rate	Human	German
	2. DNA isolated from blood lack PD-1.3G/A polymorphism (+ 7146G/A) and PD-1.5C/T polymorphism (+ 7785C/T) and also SNP’s in intron 4 and exon-5 in PDCD1 gene	2. Co-occurance of PD-1.5 T allele with CTLA4 + 49 AA homozygosity was reduced among the patients. Apart, no obvious role of PD-1 in GPA and ANCA-associated GPA	Human	Swedish, Dutch and Caucasians
CSV [36]	DNA isolated from blood lack PD-1.3G/A polymorphism (+ 7146G/A) and PD-1.5C/T polymorphism (+ 7785C/T) in PDCD1 gene	No obvious role of PD-1 in CSV and ANCA-associated CSV	Human	Dutch and Caucasians

GCA: Giant cell arteritis; KD: Kawasaki disease; BD: Beycet’s disease; GPA: Granulomatosis with polyangiitis; fWG: formerly Wegener’s granulomatosis; CSV: Churg–Strauss vasculitis; t: transcripts; p: protein; SNP: single nucleotide polymorphism; CMV: cytomegalovirus; ANCA: antineutrophilic cytoplasmic antibody; PBMCs: peripheral blood mononuclear cells; DCs: dendritic cells.

**Table 2:** Conditional Expression of PD-1 and PD-L1 on different localized endothelial cells.

Endothelial Cell Origin and References	Basal Expression of PD-L1/PD-1	Inflamed Expression of PD-L1/PD-1	Species Studied
Corneal EC [84]	Constitutive PD-L1 expression	Enhanced expression by IFN-γ	Human
Corneal EC [85]	No PD-L1 expression	Enhanced PD-L1 expression after electrocautery	Mouse
Lung and heart EC, microvascular EC line MS-1 [86]	Constitutive expression of PD-L1	Not investigated	Mouse
Infantile haemangiomas and venous malformalies ECs [87]	No PD-L1 expression High PD-1 expression	Not investigated	Human
Skin tissue EC [87]	No PD-L1 and PD-1 expression	Not investigated	Human
Microvascular pancreatic ECs [70]	Constitutive expression	Enhanced PD-L1 expression by IFN-α, -β and -γ	Mouse
Lung, heart, pancreas and stomach ECs [70]	Constitutive expression	Not investigated	Mouse
Brain tissue EC [70]	Not investigated	Enhanced PD-L1 expression by IL-12	Mouse
Liver sinuid EC [88]	Basal PD-L1 expression	Enhanced PD-L1 expression in sepsis model of induced peritonitis	Mouse
Lung EC [89]	Basal PD-L1 expression	Enhanced PD-L1 expression in hemorrhagic shock model	Mouse
Lymphatic EC [90]	Basal expression of PD-L1	Not investigated	Mouse
HUVEC [73]	No basal PD-L1 expression	Enhanced by IFN-γ and TNF-α	Human
Heart EC [73]	No basal PD-L1 expression	Enhanced by IFN-γ and TNF-α	Mouse
Heart EC [91]	No basal PD-L1 expression	Enhanced after in vivo activation by CD8^+^ T cells	Mouse
HUVEC [92]	Low basal PD-L1 expression	Enhanced by IFN-y and -α, TNF-α, CD4^+^ T cells	Human
Brain EC [93]	No basal expression	Enhanced by TNF-α and IFN-γ	Human
HUVEC [94]	No basal expression	Enhanced by IFN-γ but not by TNF-α	Human
HUVEC [95]	No basal expression	Enhanced by coculture with CD4^+^CD25^+^foxp3^+^ regulatory T cells	Human
Heart and Brain EC [96]	Basal PD-L1 expression in heart EC	Enhanced PD-L1 expression on brain ECs in vivo by autoimmune encephalomyelitis	Mouse

EC: Endothelial Cell; HUVEC: Human Umbilical Vascular Endothelial Cells; Foxp3: Forkhead box 3; IFN-γ: Interferon-gamma; TNF-α: Tumor Necrosis Factor-alpha.
